# Implementation of the “No ICU – Unless” approach in postoperative neurosurgical management in times of COVID-19

**DOI:** 10.1007/s10143-022-01851-y

**Published:** 2022-09-08

**Authors:** Lina-Elisabeth Qasem, Ali Al-Hilou, Kai Zacharowski, Moritz Funke, Ulrich Strouhal, Sarah C. Reitz, Daniel Jussen, Marie Thérèse Forster, Juergen Konczalla, Vincent Matthias Prinz, Kristin Lucia, Marcus Czabanka

**Affiliations:** 1 Department of Neurosurgery, University Hospital Frankfurt, Frankfurt, Germany; 2 Departments of Anaesthesiology, Intensive Care Medicine and Pain Therapy, University Hospital Frankfurt, Frankfurt, Germany; 3 Department of Neurology, University Hospital Frankfurt, Frankfurt, Germany

**Keywords:** ICU, Postoperative care, Neurosurgery, COVID-19 pandemic, Elective craniotomy

## Abstract

Following elective craniotomy, patients routinely receive 24-h monitoring in an intensive care unit (ICU). However, the benefit of intensive care monitoring and treatment in these patients is discussed controversially. This study aimed to evaluate the complication profile of a “No ICU – Unless” strategy and to compare this strategy with the standardized management of post-craniotomy patients in the ICU. Two postoperative management strategies were compared in a matched-pair analysis: The first cohort included patients who were managed in the normal ward postoperatively (“No ICU – Unless” group). The second cohort contained patients routinely admitted to the ICU (control group). Outcome parameters contained detailed complication profile, length of hospital and ICU stay, duration to first postoperative mobilization, number of unplanned imaging before scheduled postoperative imaging, number and type of intensive care interventions, as well as pre- and postoperative modified Rankin scale (mRS). Patient characteristics and clinical course were analyzed using electronic medical records. The No ICU – Unless (NIU) group consisted of 96 patients, and the control group consisted of 75 patients. Complication rates were comparable in both cohorts (16% in the NIU group vs. 17% in the control group; *p* = 0.123). Groups did not differ significantly in any of the outcome parameters examined. The length of hospital stay was shorter in the NIU group but did not reach statistical significance (average 5.8 vs. 6.8 days; *p* = 0.481). There was no significant change in the distribution of preoperative (*p* = 0.960) and postoperative (*p* = 0.425) mRS scores in the NIU and control groups. Routine postoperative ICU management does not reduce postoperative complications and does not affect the surgical outcome of patients after elective craniotomies. Most postoperative complications are detected after a 24-h observation period. This approach may represent a potential strategy to prevent the overutilization of ICU capacities while maintaining sufficient postoperative care for neurosurgical patients.

## Introduction

Following elective craniotomy, neurosurgical patients are routinely admitted to the ICU for a 24-h observation period to rule out early postoperative complications. These are thought to most commonly occur on the first postoperative day and include hemorrhage, liquor circulation disorders, seizures, and acute ischemic strokes [6]. Postoperative care in the ICU allows rapid recognition of neurologic deterioration or accurate hemodynamic assessment. However, there is no scientific evidence for the benefit of intensive medical monitoring in patients after elective intracranial procedures [5–7]. More likely, only a small portion of neurosurgical patients require invasive monitoring and intensive care interventions [3, 6]. Especially during the coronavirus disease-19 (COVID-19) pandemic, ICU capacities have become a critical medical resource with increasing relevance for the routine postoperative ICU care of neurosurgical patients [1, 10, 11].

Consequently, this study aimed to compare the concept of routine ICU admission in patients undergoing elective craniotomy with the concept of providing intensive care only for selected cases with potentially complicated courses during restrictions to ICU capacities and personnel induced by the COVID-19 pandemic.

## Methods

### Patients

We retrospectively identified all patients aged 18 years and older undergoing elective craniotomy and transnasal/transsphenoidal resection in our institution between February and August 2021.

The control group consisted of adult patients (> 18 years old) undergoing elective craniotomies as well as transnasal/transsphenoidal approaches between February and May 2021. During this period, all patients undergoing elective craniotomies were referred to the ICU postoperatively for 24 h as standard procedure.

From May 2021, the standard procedure for postoperative monitoring in our institute was changed to the “No ICU – Unless” concept as published previously [7]. Patients in this “No ICU – Unless” group (NIU group) were automatically planned for postoperative observation in the neurosurgical normal ward following a short period (approximately 1–2 h) in the post-anesthesia care unit unless one or more of the following preoperative criteria were fulfilled (No ICU – Unless criteria). In this scenario, patients were planned for postoperative management in the ICU:

1. Neurological and neurosurgical criteria: Tumors of the posterior fossa larger than 3 cm diameter; affection of lower cranial nerves with (potential for) dysphagia and aspiration; altered level of consciousness before surgery.
2. Anesthesiologic criteria: Cardiopulmonary or hemodynamic risk factors determined by the managing anesthesiologist, such as an American Society of Anesthesiology (ASA) score of 4 or higher, coagulation disorders, or difficult airways.

In case of high intraoperative blood loss (more than two liters), the occurrence of intraoperative complications, prolonged surgical time (more than four hours) or other anesthesiologic concerns [6], patients were admitted to the ICU even if they were initially intended for the normal ward.

Patients under 18 years of age and those undergoing emergency treatment were excluded from our analysis. Also, patients undergoing tumor biopsies were not included in our analysis.

### Data collection

Electronic medical records were used to collect patient demographics, anesthesiology documentation, intraoperative, and imaging data. In particular, we recorded surgical time, catecholamine doses, blood loss, intraoperative complications (surgical complications, seizures, intraoperative neuromonitoring break-offs, cardiac complications), total length of stay in the hospital, length of stay in the ICU, time to first postoperative mobilization, and numbers of unscheduled imaging prior to scheduled postoperative imaging. When applicable, we recorded ICU treatment received (blood pressure medication/circulation support, prolonged ventilation, observation/monitoring alone).

Finally, postoperative complications were recorded in detail. Clinically significant complications were determined as hemorrhage, acute ischemic stroke, seizure, edema, hydrocephalus, pulmonary embolism, cerebrospinal fluid (CSF) fistula, or other complications that required surgery, (re-) transfer to the ICU, or any medical intervention. Time of occurrence of complications was also noted during the period of hospital stay. Unexpected events that did not require treatment or had no practical consequences were not included in the analysis. Only in-hospital complications were analyzed.

### Statistical analysis

Data analysis was performed with Excel (version 14.7.7; Microsoft) and SPSS (version 28.0; IBM Corp). Group comparisons were performed using the Mann–Whitney U test. Fisher’s exact test was used for the comparison of categorical variables. Results of *p* ≤ 0.05 were considered statistically significant.

## Results

### Patient characteristics

In total, 171 patients were evaluated between February and August 2021. A total of 96 patients were included in the NIU group, and 75 patients were referred to the control group.

The median age of the NIU and control groups was 55 years (range of NIU group: 19–81 years; range of control group: 24–81 years; *p* = 0.171). The NIU group included 57 male (59%) and 39 (41%) female patients, and the control group contained 32 (43%) male and 43 (57%) female patients (*p* = 0.083).

The NIU group contained more patients (9%) with an ASA score of 4 versus 3% in the control group, which was not statistically significant (*p* = 0.055). The most common diagnosis in both groups was an intracranial tumor (84% in the NIU group, 81% in the control group), most commonly with a supratentorial localization (88% in both groups), followed by vascular pathologies (12% in the NIU group, 9% in the control group). No statistically significant differences in the distribution of age, gender, ASA score, diagnosis, and localization of the lesion were detected between groups (Table 1).

**Table 1:** Patient characteristics

	NIU group	Control group	*p*
Age in years Median (range)	55 (19–81)	55 (24–81)	0.171
Gender Male Female	57 (59%)39 (41%)	32 (43%) 43 (57%)	0.083
ASA score 1 2 3 4	1 (1%) 35 (37%) 51 (53%) 9 (9%)	7 (9%) 25 (33%) 41 (55%) 2 (3%)	0.055
Diagnosis Tumor Vascular Infectious Epilepsy	81 (84%) 11 (12%) 2 (2%) 2 (2%)	61 (81%) 7 (9%) 2 (3%) 5 (7%)	0.476
Localization Supratentorial Infratentorial	85 (88%) 11 (12%)	66 (88%) 9 (12%)	0.817
Total (*n*)	96	75	

Values are given either as median and range or as total and percentage of the total cohort.

*p < 0.05.

Among patients in the NIU group, 42 (44%) were transferred to the normal ward and 54 (56%) patients were admitted to the ICU; 37 patients (39%) were initially scheduled for ICU, and 17 (18%) were unscheduled, but ultimately required, transfer to the ICU as decided within the course of surgery. In the control group, 14 patients (19%) were directly admitted to the normal neurosurgical ward after surgery (resection of pituitary tumors via a transnasal/transsphenoidal approach or open biopsies) and 61 patients (81%) were admitted to the ICU (Table 2).

**Table 2:** Distribution of patients to the ICU vs. normal ward

	NIU group	Control group
ICU total ICU scheduled ICU unscheduled	54 (56%) 37/96 *(39%)* 17/96 *(18%)*	61 (81%) 61/75 *(100%)* 0 (0%)
Normal ward	42 (44%)	14 (19%)

Allocation of patients to normal ward vs. ICU in the “No ICU – Unless” group and the control group. Non-italic values in parentheses are % of the total of all subgroups; values in italics indicate the percentage within the subgroups ICU or normal ward.

**Fig. 1:**
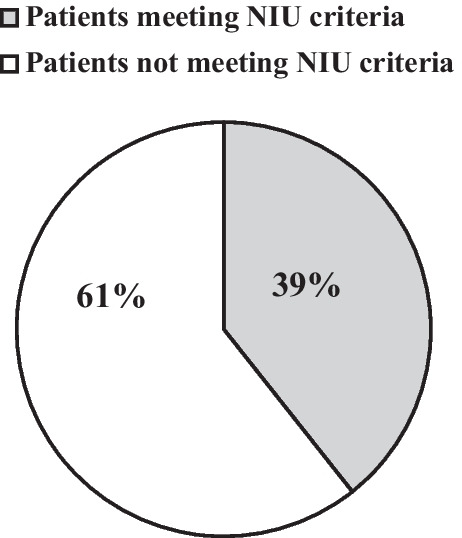
Application of No ICU – Unless criteria in the control group. The figure represents the proportions of patients in the control group which would have met and not met the NIU criteria; 39% of patients would have met the NIU criteria for observation in the ICU versus 61% who could have been observed in the normal ward

Retrospective analysis of the control group showed that 39% of patients would have fulfilled the NIU criteria for observation in the ICU, with 61% having been transferred to the normal ward instead of ICU observation (Fig. 1).

### Intraoperative parameters

Intraoperative parameters in the NIU and control groups were examined (Table 3). Median length of surgery in minutes (157; range 21–358 vs. 181; range 279–470; *p* = 0.369), blood loss in milliliters (300; range 10–1500 vs. 325; range 10–1000; *p* = 0.726), intraoperative catecholamines in µg/kg/min (0.06; range 0.00–0.20 vs. 0.08; range 0.00–0.25; *p* = 0.840), and number of intraoperative complications (8/96; 8% vs. 3/75; 4%; *p* = 0.73). Intraoperative complications occurring in the NIU group included cardiac arrhythmia in five cases (0,05%), vessel injury or aneurysm rupture in two cases (0,02%), and one intraoperative seizure (0,01%). Intraoperative complications within the control group included cardiac arrhythmia in two cases (0,03%) and loss of intraoperative neuromonitoring signals in one case (0,01%). All intraoperative parameters showed no statistically significant differences (Table 3).

**Table 3:** Intraoperative parameters

	NIU group	Control group	*p*
Length of surgery (min) Median (range)	157 (21–358)	181 (29–470)	0.369
Blood loss (ml) Median (range)	300 (10–1500)	325 (10–1000)	0.716
Intraoperative catecholamines (µg/kg/min) Median (range)	0.06 (0.00–0.20)	0.08 (0.00–0.25)	0.840
Intraoperative complications Yes No	8 (8%) 88 (92%)	3 (4%) 72 (96%)	0.073

Values are given either as median and range or as a percentage of the total cohort.

*p < 0.05.

### Inpatient stay

#### Overall length of stay in hospital

Patients in the NIU group had an overall shorter stay in the hospital compared to the control group (average 5.8 ± 4.6 vs. 6.8 ± 3.7 days; *p* = 0.661; Table 4). Patients from the NIU group whose admission to the ICU was unscheduled had an overall hospital stay of 6.0 (± 4.7) days which was comparable to the subgroup of NIU patients that were scheduled for postoperative ICU admission (average 6.1 ± 4.7; *p* = 0.571; Table 4).

**Table 4:** In-hospital measures

	Days in hospital, average (standard deviation)	Days in ICU, average (standard deviation)	Days to first mobilization, average (standard deviation)	Number of unplanned imaging scans, no. (% of cohort)
NIU group				
Total ICU scheduled ICU unscheduled Normal ward	5.8 (± 4.6) 6.0 (± 4.8) 5.8 (± 4.6) 5.8 (± 4.6)	0.9 (± 1.8) 1.0 (± 1.9) 1.0 (± 1.9) -	0.9 (± 1.6) 1.0 (± 1.6) 1.0 (± 1.7) 1.0 (± 1.8)	10 (10%) 6 (6%) 3 (3%) 1 (1%)
Control group Total	6.8 (± 3.7)	1.3 (± 2.1)	1.1 (± 1.2)	10 (13%)
P	0,661	0,414	0,402	0,671

Days in hospital and as applicable days on ICU for the “No ICU – Unless” group and the control group as well as days until first mobilization (values as average and standard deviation). Numbers of unplanned CT or MRI scans are given as absolute counts.

*NIU*, No ICU – Unless; *CT*, computed tomography; *MRI*, magnetic resonance imaging.

#### Length of stay in ICU

Overall, patients in the NIU group spent less time in the ICU following surgery than the control group (average 0.9 ± 1.8 vs. 1.4 ± 2.1 days; *p* = 0.414; Table 3). Patients from the NIU group who were scheduled for ICU admission following surgery spent the same time in the ICU as those with unscheduled admission (average 1 ± 1.9 days; *p* = 0.667; Table 4).

#### Time to first postoperative mobilization

There was no difference between groups in time to mobilization (average 0.9 ± 1.6 days in the NIU group vs. 1.1 ± 1.2 days in the control group; *p* = 0.402; Table 4).

#### Number of unscheduled imaging

11/96 patients (11%) received unscheduled imaging (computed tomography (CT) or magnetic resonance imaging (MRI)) within the NIU group prior to their otherwise scheduled postoperative imaging. One patient received two CTs prior to scheduled imaging; 9/75 patients (12%) received CT scans prior to scheduled MRI or CT scan within the control group. One of these patients received two unscheduled CT scans. Within the control group, ten unscheduled scans were conducted (13%); within the NIU group, eleven scans were initiated prior to scheduled imaging (11%). Overall, cohorts did not differ significantly in the number of unplanned CT/MRI scans (*p* = 0.67; Table 4).

### ICU treatment

In the NIU group, 11/96 patients (11%) received treatment in the ICU and 43 were observed without intervention. In the control group, 15/75 patients (20%) received treatment in the ICU and 46/61 were observed without intervention.

No statistically significant differences were found between the type of interventions in ICU in the NIU group and control group: anti-hypertensive therapy in 4 (5%) patients in vs. 5 (6%) patients (*p* = 0.825); catecholamine therapy in 6 (8%) patients vs. in 3 (3%) patients (*p* = 0.591); ventilation in 5 (7%) patients vs. 3 (3%) patients (*p* = 0.552).

In both groups, intravenous blood pressure management was the most frequent type of treatment in post-craniotomy patients (Table 5).

**Table 5:** Treatment in ICU

	Oral anti-hypertensive therapy	i.v. anti-hypertensive therapy	Catecholamines	Observation/monitoring	Ventilation
NIU group Total ICU scheduled ICU unscheduled	2 (2%) 1 (1%) 1 (1%)	3 (3%) 0 (0%) 3 (3%)	3 (3%) 1 (1%) 2 (2%)	43 (45%) 32 (33%) 11 (12%)	3 (3%) 3 (3%) 0 (0%)
Control group Total	1 (1%)	3 (4%)	6 (8%)	46 (61%)	5 (7%)

The table includes only patients with admission to the ICU (excluding 14 patients from the control group and 43 patients from the “No ICU – Unless” group who were transferred directly to the normal ward postoperatively). Values given as absolute count and % of all patients within the total cohort.

*NIU*, No ICU – Unless; *i.v.*, intravenous.

### Early postoperative complications

**Fig. 2:**
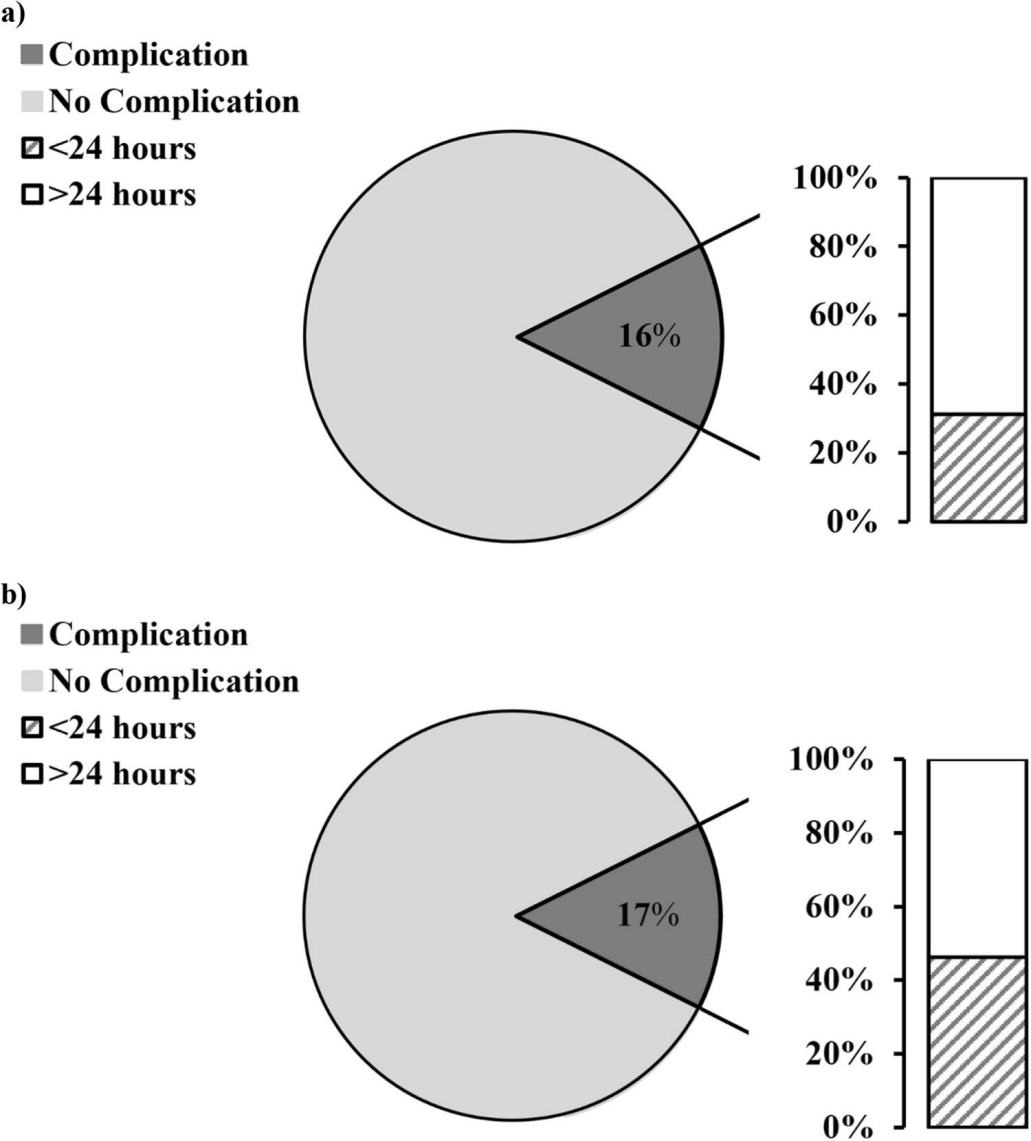
Postoperative complications.Percentage of the total cohort of postoperative complications subdivided into early (< 24 h after surgery) and late (> 24 h after surgery). (**a**) NIU group: of the 16% overall complication rate, 33% occurred within the first 24 h following surgery with the remaining complications occurring after 24 h. (**b**) Control group: the overall complication rate was 17%; 46% of these complications occurred within the first 24 h following surgery and 54% occurred after 24 h postoperatively

Incidence of early postoperative complications during the postoperative inpatient hospital stay within the NIU and control groups showed no significant differences (15/96; 16% vs. 13/75; 17%; *p* = 0.786; Fig. 2). Further analysis of the time point at which complications occurred, either before or after the first 24 h following surgery, showed that most complications in both NIU and control groups occurred after the first 24 h postoperatively (67% in the NIU group and 54% in the control group).

In the NIU group, 7/15 (46%) of the complications occurred in the normal ward, with one patient suffering two early postoperative complications (hemorrhage and stroke). In the control group, 6/13 (46%) complications occurred in the normal ward.

One patient suffered two complications during the inpatient stay (seizure and stroke).

Surgical intervention was required in 9/15 (60%) of complications occurring in the NIU group (five evacuations of hemorrhage, two implantations of external ventricular drains, and one re-resection due to space-consuming residual hemorrhaging tumor mass).

Within the control group, 3/13 (23%) complications required surgical intervention (two evacuations of hemorrhage and one re-resection due to space-consuming residual tumor mass).

A detailed summary of all postoperative complications is found in Table 6 and Table 7.

**Table 6:** Detailed summary of early postoperative complications in the “No ICU – Unless” group

Patient no.	Diagnosis	Complication	Time point (hours postop)	Ward	Clinical presentation	Imaging (hours postop)	Therapy	mRS at admission	mRS at discharge
4	GBM	Seizure (grand mal)	12 h	ICU	Reduced consciousness, saturation drop	CT 12 h	Re-intubation; anticonvulsive Tx	1	1
9	Meningioma	Seizure (non-convulsive) + edema	0 h	ICU	Aphasia/dysphasia	-	Anticonvulsive Tx; hyperosmolar meds	0	0
11	Astrocytoma	Hemorrhage (EDH) + edema	1 h	ICU	Intraoperative SSEP loss	CT 1 h	Surgery	5	4
17	GBM	Thrombocytopenia	24 h	NW	None	-	Transfer to ICU; platelet substitution	1	1
22	Meningioma	Pulmonal embolism	72 h	ICU	Saturation drop, bradycardia	CT 72 h	Anticoagulation	1	1
26	Meningioma	Hemorrhage (ICH)	10 h	ICU	Reduced consciousness	CT 10 h	EVD	1	1
36	Metastasis	Hydrocephalus	48 h	NW	Reduced consciousness, ataxia	MRT 48 h	EVD	2	2
41	Metastasis	Hemorrhage (ICH)	15 h	ICU	Reduced consciousness	CT 16 h	Surgery	1	6
42	Meningioma	Hemorrhage (EDH)	24 h (incidential) 72 h (progress)	NW	Aphasia	MRT 24 h CT 72 h	Surgery	1	1
53	GBM	Hemorrhage (EDH)	48 h (incidential)	NW	None	-	Surgery	0	0
59	dAVF	Hemorrhage (ICH)	37 h (incidential)	NW	None	-	None	1	1
66	GBM	Stroke + hemorrhage (ICH)	Stroke: 0 h ICB: 24 h	ICU	Hemiparesis + aphasia	CT 2 h	Surgery	1	5
73	Ganglioglioma	Hemorrhage (EDH)	96 h (incidential)	NW	None	-	None	2	2
94	GBM	Hemorrhage (EDH)	24 h	NW	Hemiparesis	MRT 24 h	Surgery	3	3

Diagnosis, underlying surgery, description of complications and their clinical manifestations, the timepoint, and ward on which the patient was at the time the complication was first registered (ICU vs. normal ward). If imaging was performed, the timepoint of such imaging is included as well as mRS at admission and discharge.

*CT*, computed tomography; *dAVF*, dural arteriovenous fistula; *EDH*, epidural hemorrhage; *EVD*, external ventricular drain; *ICH*, intracerebral hemorrhage; *ICU*, intensive care unit; *mRS*, modified Rankin scale; *MRT*, magnetic resonance imaging; *NW*, normal ward; *GBM*, glioblastoma multiforme.

**Table 7:** Detailed summary of early postoperative complications in the control group

Patient no.	Diagnosis	Complication	Time point (hours postop)	Ward	Clinical presentation	Imaging (hours postop)	Therapy	mRS at admission	mRS at discharge
98	Meningioma	Hemorrhage (ICH)	3 h	ICU	Aphasia	CT 3 h	None	2	2
100	Pituitary adenoma	Residual tumor volume	26 h	NW	Oculomotor and abducens nerve palsy	MRT 28 h	Surgical resection	2	2
101	Aneurysm	Stroke (Heubner infarct) + seizure (non-convulsive)	Stroke: 0 h Seizure: 29 h	Stroke: ICU Seizure: NW	Aphasia, Todd’s paresis	MRT 5 h; CT 29 h	Anticonvulsive Tx; shift to ICU	1	1
102	Empyema	Hemorrhage (ICH)	72 h	ICU	Anisocoria, reduced consciousness	CT 72 h	Therapy limitation	4	6
109	Meningioma	Hemorrhage (ICH)	72 h	NW	Aphasia, hemiparesis	CT 72 h	Surgery	4	4
113	Meningioma	Stroke	0 h	ICU	Aphasia, hemiparesis post op	CT 3 h	None	1	4
119	GBM	Pulmonal embolism	5 days	NW	DVT symptoms	CT-A 5 days	Anticoagulation; shift to IMC	1	4
124	Meningioma	Edema	0 h	ICU	Increased ICP	CT 10 h	Prolonged ventilation; hyperosmolar meds	1	2
147	Meningioma	Hemorrhage (ICH)	24 h	NW	None	CT 24 h (incidential)	Shift to ICU	0	1
157	Vestibular schwannoma	CSF fistula	24 h	NW	Oozing wound	-	Surgery	1	1
167	GBM	Seizure (non-convulsive)	19 h	ICU	Reduced consciousness	CT 19 h	Anticonvulsive Tx	1	1
164	Epidermoid	Hemorrhage (ICH)	5 h	ICU	Reduced consciousness	CT 5 h	None	1	5

Diagnosis, underlying surgery, description of complications and their clinical manifestations, the timepoint, and ward on which the patient was at the time the complication was first registered (ICU vs. normal ward). If imaging was performed, the timepoint of such imaging is included as well as mRS at admission and discharge.

*CSF*, cerebrospinal fluid; *CT*, computed tomography; *EDH*, epidural hemorrhage; *EVD*, external ventricular drain; *ICH*, intracerebral hemorrhage; *ICU*, intensive care unit; *IMC*, intermediate care unit; *mRS*, modified Rankin scale; *MRT*, magnetic resonance imaging; *NW*, normal ward; *GBM*, glioblastoma multiforme.

### Pre- and postoperative mRS

**Fig. 3:**
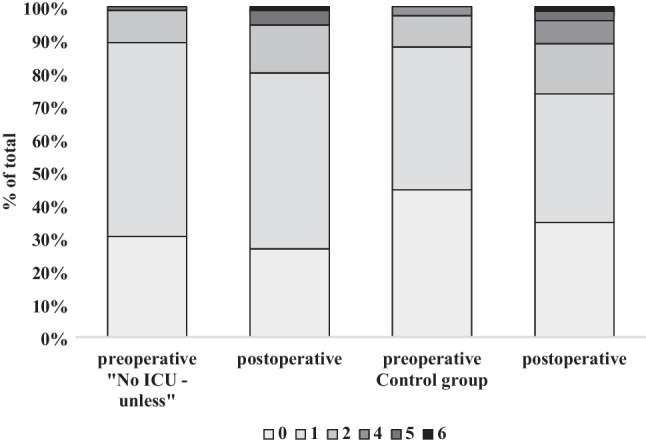
Pre- and postoperative mRS scores are presented as percent of the total in each of the No ICU – Unless and control groups at pre- and postoperative time points

There was no significant change in the distribution of preoperative (*p* = 0.960) and postoperative (*p* = 0.425) mRS scores in the NIU group and the control group with the majority of patients in both groups having a pre- and postoperative mRS score of 1 (Fig. 3).

## Discussion

Results of the current study demonstrate that postoperative management of elective cranial neurosurgical procedures is safe with a comparable complication and risk profile compared to standard postoperative ICU management. Most complications occurred later than 24 h following surgery, in which case patients are usually already on the regular ward. Outcome analysis at discharge from the hospital found that the “No ICU – Unless” concept resulted in comparable mRS distribution compared to the control group which underwent automatic postoperative ICU observation.

### Postoperative care in patients undergoing elective craniotomy

The results of this study support previous findings in screening post-craniotomy patients for the need for ICU admission. Bui et al. [3] compared routine ICU admission to normal ward admission in 343 patients after elective craniotomy and identified long duration of surgery, anesthesiologic risks, and high intraoperative blood loss as predictors for required ICU admission. A further retrospective study including 400 patients identified advanced age and diabetes as independent predictors for necessary postoperative ICU monitoring [6]. Overall, no study could reveal routine ICU admission in all postoperative patients as significantly beneficial for in-hospital morbidity and mortality [2–4, 6, 7].

Despite the reduction of automatic postoperative transfers to the ICU within the NIU group, implementing the “No ICU – Unless” concept does not entirely exclude the a priori allocation of selected patients to the ICU following surgery. To date, evidence-based solid criteria that can effectively identify patients who would most profit from postoperative ICU observation do not exist. Our study identifies patients who will require ICU observation based on pragmatic criteria which can guide neurosurgeons and anesthesiologists in daily practice.

First, we initiate ICU observation in patients undergoing resection of large infratentorial lesions, as the previous series have shown that up to 43% of these patients required re-transfer to the ICU from the normal ward and approximately 4% required re-intubation [2]. Furthermore, lesions affecting lower cranial nerves may promote dysphagia and aspiration, so we routinely transfer patients with such lesions to the ICU [6]. Finally, patients with altered levels of consciousness prior to surgery were also routinely transferred to the ICU to observe and react to persisting or worsening neurological deficits.

In our study, the common reasons for unscheduled ICU admission within our “No ICU – Unless” group included intraoperative complications (8/96 patients) and unforeseeable prolonged surgical time (4/96 patients). Hanak et al. [6] identified intravenous blood pressure management as the most frequent type of ICU treatment modality, as it was also found in both of our cohorts. Most patients received observation and monitoring alone after being admitted to the ICU (45% of all patients within the NIU group).

### Early postoperative complications

In our analysis, most complications occurred after the transfer of the patient to the normal ward after 24 h of observation (67%). More importantly, the incidence of complications did not increase after introducing the “No ICU – Unless” policy (16% in the NIU group vs. 17% in the control group; *p* = 0.786). These observations are in line with previously reported data on postoperative complications [8]. Importantly, no increase in complications has been described when patients were managed in the normal ward after surgery [7]. One study reported a statistically significant reduction of the complication rate from 0.98 to 0.53 per patient after changing the management of post-craniotomy patients to a normal ward setting [7].

The most common postoperative complication requiring surgical intervention in both the NIU and control groups was postoperative hematoma (9% in the NIU group and 7% in the control group; in both groups, 7% of all hematomas required surgical intervention).

Between both groups, three hemorrhages occurred within the first 24 h after surgery. The rate of postoperative hematoma following elective craniotomy has been reported to reach 1–4%; of these, only 2,1% required operative evacuation in the first 30 days after surgery [8]. Analysis of a series of 50 postoperative hematomas [12] revealed that 44 occurred within the first six hours after surgery. Six (12%) hemorrhages occurred later than 24 h. A further study found 16% of neurologic complications in 168 post-craniotomy patients, with 85% of complications occurring in the first two hours after surgery [9].

Taken together, 24-h observation and monitoring in the ICU to rule out early postoperative complications, most likely hemorrhage, may not be considered effective as only 1,7% of postoperative hemorrhages in our study occurred within 24 h. Observation up to a 6-h recovery room may also be sufficient [1–3, 5, 7, 8, 10, 11].

### ICU capacity in times of COVID-19

According to the German Intensive Care Unit Register (DIVI), the capacity of available ICU beds in Germany decreased from approximately 12.000 at the beginning of the pandemic in March 2020 to approximately 2.200 during the fourth wave of the pandemic in November 2021 [4]. As this pandemic is ongoing, strategies for ICU capacity management are more critical than ever. The concept of “No ICU – Unless” may therefore not only serve as a clinically relevant postsurgical management strategy for neurosurgical patients but may also assist in relieving strain on the healthcare system.

Limitations of our study include the single-center, retrospective design, limiting the ability to generalize these initial results to other centers. Further studies should include a multicentric evaluation of the “No ICU – Unless” concept and include long-term follow-up in centers which have also been heavily impacted by pandemic-associated limitations in order to validate the results of our single-center analysis.

## Conclusion

We found that routine postoperative ICU management does not reduce postoperative complications and has no effect on the surgical outcome of patients after elective craniotomies. Most postoperative complications were detected after a 24-h observation period. This approach may represent a potential strategy to prevent the overutilization of ICU capacities while maintaining sufficient postoperative care for neurosurgical patients. In our experience, the preoperative assessment of patients for postoperative ICU admission should be a team-based decision of experienced neurosurgeons and anesthesiologists in which comorbidities should be considered.

## Data Availability

Not applicable.
