# Correction to: Implementation of the “No ICU – Unless” approach in postoperative neurosurgical management in times of COVID‑19

**DOI:** 10.1007/s10143-023-01959-9

**Published:** 2023-02-10

**Authors:** Lina‑Elisabeth Qasem, Ali Al‑Hilou, Kai Zacharowski, Moritz Funke, Ulrich Strouhal, Sarah C. Reitz, Daniel Jussen, Marie Thérèse Forster, Juergen Konczalla, Vincent Matthias Prinz, Kristin Lucia, Marcus Czabanka

**Affiliations:** 1 Department of Neurosurgery, University Hospital Frankfurt, Frankfurt, Germany; 2 Departments of Anaesthesiology, Intensive Care Medicine and Pain Therapy, University Hospital Frankfurt, Frankfurt, Germany; 3 Department of Neurology, University Hospital Frankfurt, Frankfurt, Germany

**Correction to: Neurosurgical Review (2022) 45:3437–3446**

10.1007/s10143-022-01851-y

The authors regret that in the original article Kristin Lucia and Marcus Czabanka were recognized as shared corresponding authors, however the status of co-senior author was not noted. As such, Kristin Lucia and Marcus Czabanka are both co-corresponding and co-senior authors.

The original article has been corrected.

